# Osteosarcoma incidence in dogs following a tibial plateau levelling osteotomy

**DOI:** 10.18849/ve.v10i4.720

**Published:** 2025-10-23

**Authors:** Laura Sweeting+, Kenneth Johnson+

**Keywords:** BONE PATHOLOGY, CANINE, ORTHOPAEDIC DISEASE, OSTEOSARCOMA, TIBIAL PLATEUA LEVELLING OSTEOTOMY

## Abstract

**PICO question:**

In dogs, does undergoing a tibial plateau levelling osteotomy (TPLO) compared to not undergoing a TPLO affect the future risk of developing osteosarcoma?

**Clinical bottom line:**

**Category of research:**

Risk.

**Number and type of study designs reviewed:**

One experimental study, designed as a matched case control.

**Strength of evidence:**

Weak.

**Outcomes reported:**

Only one study is appropriately designed to investigate a TPLO and associated osteosarcoma risk. This study found a 40-fold increased risk in dogs with a history of TPLO, compared to dogs that had not undergone a TPLO (OR, 40.65; 95% CI, 4.04 to 409.06; P = 0.002).

**Conclusion:**

There is weak yet preliminary indication to suggest a TPLO increases a dog’s risk of developing osteosarcoma later in life. However, with such a wide confidence interval, the true effect remains unclear. Additional matched case-control studies are needed to strengthen knowledge of this correlation, however until this time osteosarcoma should be considered as a differential diagnosis in post-TPLO dogs which present with lameness and/or proximal limb swelling.

## How to apply this evidence in practice

The application of evidence into practice should take into account multiple factors, not limited to: individual clinical expertise, patient’s circumstances and owners’ values, country, location or clinic where you work, the individual case in front of you, the availability of therapies and resources.

Knowledge Summaries are a resource to help reinforce or inform decision-making. They do not override the responsibility or judgement of the practitioner to do what is best for the animal in their care.

## The evidence

Selmic et al. (2018) was the only paper appropriately designed to investigate a possible tibial plateau levelling osteotomy (TPLO) and associated osteosarcoma risk. Although this one study does support an increased risk, evidence is weak due to the limited study numbers. Further matched case-controlled investigation is needed to appropriately assess a possible TPLO-osteosarcoma correlation.

## Summary of the evidence

**Selmic et al. (2018):** Osteosarcoma following tibial plateau leveling osteotomy in dogs: 29 cases (1997–2011) **Aim: **To determine if dogs that have undergone a tibial plateau leveling osteotomy (TPLO) are at an increased risk of developing proximal tibial osteosarcoma.

Population:	Dogs evaluated at the Colorado State University Veterinary Teaching Hospital (USA) between 1 January 2005 and 31 December 2012.
Sample size:	113 dogs.
Intervention details:	Case dogs (n = 34): client owned dogs with histologically confirmed proximal tibial osteosarcoma, diagnosed ≥ 1 year following a TPLO procedure (11 with prior TPLO). Control dogs (n = 79): client owned dogs that did not have a history of osteosarcoma (6 with prior TPLO). Matched to cases 3:1 (when possible) by breed, age (≤ 2 years age difference) and initial exam (≤ 1 year a part).
Study design:	Matched case control.
Outcome Studied:	Comparison of osteosarcoma incidence between cases and controls, controlling for breed, age and initial exam.
Main Findings (relevant to PICO question):	Dogs with a history of a TPLO were 40 times more likely to develop proximal tibial osteosarcoma than a dog who had not undergone a TPLO (OR 40.65; 95% CI 4.04–409.06; P = 0.002). Every increase in 1 kg of body weight was significantly associated with an 11% increase in proximal tibial osteosarcoma development (OR 1.11; 95% CI 1.03–1.20; P = 0.005).
Limitations:	Only one veterinary hospital database was used to collect subjects, which is perhaps not a reflection of the wider dog population. There was a lack of statistical power analysis and justification for the selection of an 8-year data collection period. The study did not control for implanted TPLO plate material type. Although unlikely, dogs in the control group may have had undiagnosed osteosarcoma (misclassification bias).

## Appraisal, application and reflection

Selmic et al. (2018) required histologic confirmation of osteosarcoma for inclusion which is accepted as an accurate diagnostic technique (Sabattini et al., 2017). It also required an osteosarcoma diagnosis of more than or equal to one year after a tibial plateau levelling osteotomy (TPLO), avoiding the possibility of pre-existing sarcomas though not eliminating the possibility that dogs were misclassified as a control. Despite this possibility for misclassification bias and its distorting effect on risk analysis, the likelihood of misdiagnosis is low. Canine studies show increased appendicular osteosarcoma incidence at weight-bearing regions of long bones, often presenting with lameness and swelling and possibly pain and pathological fractures (Szewczyk et al., 2014; Boerman et al., 2012). The aggressive nature of osteosarcoma and its conspicuous clinical signs support an accurate and timely diagnosis, allowing for reliable classification of subjects as either affected or osteosarcoma-free.

The value of Selmic et al. (2018) to this investigation is largely dictated by its matched case control study design. There are two alternate existing studies claiming to investigate an osteosarcoma and TPLO correlation, however, they are inappropriately designed to do so. These include the Sartor et al. (2014) investigation, as it lacks an essential non-TPLO exposure group to be a useful cohort study. Selmic et al. (2014) is also unsuitable because as a retrospective case series, it inherently lacks a control group (non-TPLO canines) amongst other useful features such as strong rigor and patient follow up (Sayre et al., 2017). As this Knowledge Summary intends to investigate variances in osteosarcoma incidence between dogs which have had a TPLO to those that have not, a non-TPLO control group is essential to effectively answer the PICO question. This is particularly important given that, although uncommon, spontaneous osteosarcoma has been reported at the proximal tibia (Morello et al., 2011). Although these studies (Sartor et al., 2014; Selmic et al., 2014) claim to examine a correlation between TPLO and osteosarcoma, their designs are inadequate and so highlight the need for further research to provide stronger evidence for veterinary clinicians.

Selmic et al. (2018) was insightful and valuable. As a matched case-control study, limitations of case series are removed although confounders can be introduced that did not previously exist in the data (Pearce, 2016; Rose & Van der Laan, 2009). As a result, as Rose & Van der Laan (2009) highlight, potential confounders must be accounted for in statistical analysis to maintain validity and increase precision. This was effectively performed by Selmic et al. (2018) through a multivariable conditional logistic regression model. Dog weight is a potential confounder, as studies report that large breeds are at increased risk of cranial cruciate ligament rupture (Powers et al., 2005; Whitehair et al., 1993), as well as osteosarcoma development (Wilk & Zabielska-Koczywas, 2021; Simpson et al., 2017; Szewczyk et al., 2014). Matching and statistical adjustment for weight ensures the findings reflect an investigation of a true TPLO-osteosarcoma association. Most notably, after adjusting for weight, the study found a significant 40-fold increased risk of developing proximal tibial osteosarcoma in TPLO dogs, compared to non-TPLO dogs (OR 40.65; 95% CI 4.04 to 409.06; P = 0.002). This is a highly important finding, however the extremely wide confidence interval must be noted. It highlights an uncertainty over the true effect size of a TPLO on osteosarcoma risk. Additionally, this 40-fold increase is reported as a relative risk, with no absolute risk provided. Absolute risk is necessary to better assess the true clinical significance of the TPLO procedure on osteosarcoma development. These considerations call for additional worthwhile study to be conducted to substantiate results and inform evidence-based practice. Corroboration of or building on findings in Selmic et al. (2018) could guide the implementation of follow-up screening programs for TPLO patients to promote early osteosarcoma detection, or prompt further investigation into specific TPLO characteristics such as plate material or post-operative infection.

Exploring possibilities for further study, Akobeng (2005) demonstrates that according to the hierarchy of evidence, randomised controlled trials are the best practice for an investigation of causal relationships, though this study type could prove contextually complex. Tibial plateau levelling osteotomy intervention in dogs would be compared to an alternative, ideally no surgical intervention, posing possible ethical and logistic concerns for owners. The TPLO could be compared to another accepted surgical intervention such as tibial tuberosity advancement (Trisciuzzi et al., 2019), though without solid understanding of the mechanism by which TPLOs could be increasing osteosarcoma risk, utilising two different surgical procedures as interventions could confound results. Large scale canine patient participation and informed client consent also pose practical and ethical obstructions to a randomised controlled trial (Nardini, 2014). Observational studies could strike a balance between feasibility and rigour. An emphasis could be placed on conducting matched case-controls, suited to the investigation of a single disease outcome (Gilmartin-Thomas et al., 2018). The use of statistical power analysis is a potential improvement to the matched case-control study design in Selmic et al. (2018), as well as the inclusion of only deceased patients with documented past medical histories to avoid undiagnosed osteosarcoma. A retrospective cohort study design provides another observational design option, allowing investigation of a possible TPLO-osteosarcoma causal effect by using past medical patient records, and requiring similar statistical adjustment for confounders (Setia, 2016). A TPLO and non-TPLO exposure group must be included, improving upon the cohort study design intended by Sartor et al. (2014).

An obvious limitation of this Knowledge Summary to prompt evidence-based research, arises from the fact only one paper was found to appropriately investigate the PICO question. However, there remains preliminary evidence to suggest that a higher osteosarcoma incidence is seen in dogs which have undergone a TPLO in comparison to dogs which have not. Knowledge of such a correlation between TPLO and subsequent osteosarcoma is imperative due to its aggressive nature and poor prognosis. Should a definitive correlation be established, it could also guide further study into potential carcinogenic components of the TPLO procedure. Slocum cast plates have been implicated in several case reports of osteosarcoma following a TPLO, with their high ferrite composition being a speculated carcinogen (Harasen & Simko, 2008; Boudrieau et al., 2005). However, TPLO wrought plates have also been associated with osteosarcoma cases and so alternative hypotheses are postulated, including the plate’s protraction of chronic inflammation (Atherton & Arthurs, 2012).

Tibial plateau levelling osteotomiess are veterinarians’ treatment of choice for CrCL ruptures (Von Pfeil et al., 2018) and with dog ownership increasing around the world (Larkin, 2024; Animal Medicines Australia, 2021) and CrCL ruptures a common canine orthopaedic presentation (Spinella et al., 2021; Coletti et al., 2014), TPLO surgeries will continue to be an imperative treatment in orthopaedic veterinary medicine. Thus, the industry has a responsibility to develop understanding of potential risks that accompany a veterinarian’s TPLO recommendation.

## Methodology

**Search Strategy:** 

Databases searched and dates covered:	CAB Abstracts via Web of Science 1973 – 2024 PubMed via NCBI 1966 – 2024 Scopus via Elsevier 1966 – 2024
Search strategy:	CAB Abstracts: (dog OR dogs OR canine OR canines OR canis) AND (tplo OR “tibial plateau levelling osteotomy”) AND (osteosarcoma OR “osteogenic sarcoma” OR sarcoma OR neoplasia OR neoplasm OR neoplastic) PubMed: ((dog[Title/Abstract] OR dogs[Title/Abstract] OR canine[Title/Abstract] OR canines[Title/Abstract] OR Canis[Title/Abstract]) AND (TPLO[Title/Abstract] OR "tibial plateau levelling osteotomy"[Title/Abstract] OR tibial plateau levelling osteotomy[Title/Abstract]) AND (osteosarcoma[Title/Abstract] OR "osteogenic sarcoma"[Title/Abstract] OR sarcoma[Title/Abstract] OR neoplasia[Title/Abstract] OR neoplasm[Title/Abstract] OR neoplastic[Title/Abstract])) Scopus: TITLE-ABS-KEY ((dog OR dogs OR canine OR canines OR canis) AND (tplo OR “tibial plateau levelling osteotomy”) AND (osteosarcoma OR “osteogenic sarcoma” OR sarcoma OR neoplasia OR neoplasm OR neoplastic))
Dates searches performed:	08 Nov 2024

**Exclusion / Inclusion Criteria:** 

Exclusion:	A single case study, not peer-reviewed, not relevant to the PICO question, a duplicate from a previously searched database.
Inclusion:	Any primary study written in English pertaining to osteosarcoma incidence comparison in dogs who have and have not undergone a TPLO.

**Search Outcome:** 

Database	Number of results	Excluded – single case report	Excluded – not peer reviewed	Excluded – not relevant to the PICO	Total relevant papers
CAB Abstracts	13	6	2	4	1
PubMed	10	5	0	4	1
Scopus	16	7	2	6	1
Total relevant papers when duplicates removed					1

## Acknowledgements

This manuscript was completed in partial fulfilment of requirements of the Bachelor of Veterinary Biology / Doctor of Veterinary Medicine, The University of Sydney.

## ORCiD

Laura Sweeting: https://orcid.org/0009-0003-5277-6003

Kenneth Johnson: https://orcid.org/0000-0001-6207-3924

## Conflict of Interest

The author declares no conflicts of interest.
